# Longitudinal subtypes of disordered gambling in young adults identified using mixed modeling

**DOI:** 10.1016/j.pnpbp.2019.109799

**Published:** 2020-03-08

**Authors:** Samuel R. Chamberlain, Jan Stochl, Jon E. Grant

**Affiliations:** a Department of Psychiatry, University of Cambridge, UK; b Department of Kinanthropology, Charles University in Prague, Czechia; c Department of Psychiatry & Behavioral Neuroscience, University of Chicago, United States of America; d Cambridge and Peterborough NHS Foundation Trust, UK

**Keywords:** Addiction, Cognition, Gambling, Impulsivity, Latent, Subtypes

## Abstract

**Objective:**

While many individuals gamble responsibly, some develop maladaptive symptoms of a gambling disorder. Gambling problems often first occur in young people, yet little is known about the longitudinal course of such symptoms and whether this course can be predicted. The aim of this study was to identify latent subtypes of disordered gambling based on symptom presentation and identify predictors of persisting gambling symptoms over time.

**Methods:**

575 non-treatment seeking young adults (mean age [SD] = 22.3 [3.6] years; 376 (65.4%) male) were assessed at baseline and annually, over three years, using measures of gambling severity. Latent subtypes of gambling symptoms were identified using latent mixture modeling. Baseline differences were characterized using analysis of variance and binary logistic regression respectively.

**Results:**

Three longitudinal phenotypes of disordered gambling were identified: high harm group (*N* = 5.6%) who had moderate-severe gambling disorder at baseline and remained symptomatic at follow-up; intermediate harm group (19.5%) who had problem gambling reducing over time; and low harm group (75.0%) who were essentially asymptomatic. Compared to the low harm group, the other two groups had worse baseline quality of life, elevated occurrence of other mental disorders and substance use, higher body mass indices, and higher impulsivity, compulsivity, and cognitive deficits. Approximately 5% of the total sample showed worsening of gambling symptoms over time, and this rate did not differ significantly between the groups.

**Conclusions:**

Three subtypes of disordered gambling were found, based on longitudinal symptom data. Even the intermediate gambling group had a profundity of psychopathological and untoward physical health associations. Our data indicate the need for large-scale international collaborations to identify predictors of clinical worsening in people who gamble, across the full range of baseline symptom severity from minimal to full endorsement of current diagnostic criteria for gambling disorder.

## Highlights

• This study found three subtypes of disordered gambling.
• Moderate-severe gambling disorder remained symptomatic at follow-up
• Obsessive-compulsive tendencies and inflexible cognition predicted worsening of symptoms over time

## 1 Introduction

Gambling is common across the globe and has taken place since ancient times. Many people are able to gamble recreationally without necessarily developing significant untoward consequences. A subset of people, however, develop Gambling Disorder, a Substance-Related and Addictive Disorder characterized by persistent maladaptive patterns of gambling behavior and functional impairment. (American Psychiatric Association, 2013) Gambling Disorder is associated with a host of untoward consequences (Sood et al., 2003) including relationship difficulties, (Morasco et al., 2006) financial issues (including bankruptcy), (Grant and Kim, 2001) and suicide risk. (Ledgerwood and Petry, 2004) Gambling Disorder is defined on the basis of meeting at least four of nine Diagnostic and Statistical Manual Version 5 (DSM-5) criteria. Examples of these diagnostic items include loss of control over gambling, difficulty cutting back, and ‘chasing losses’ (returning to gamble again after losing). However, endorsement of some but fewer than four diagnostic criteria, commonly termed ‘problem gambling’, is also clinically relevant. (Currie et al., 2012; Petry et al., 2005) A growing body of evidence shows that people with subclinical gambling problems experience significant harms as a consequence, including other mental disorders and worse quality of life. (Browne et al., 2017) Meta-analysis has evidenced high rates of mental health comorbidities both in Problem Gambling and Gambling Disorder. (Lorains et al., 2011) We previously reported that endorsement of two criteria can be associated with cognitive impairment, functional impairment, and elevated occurrence of other mental disorders similar to that observed in the full disorder. (Chamberlain et al., 2017) These findings highlight the importance of considering disordered gambling across the full range of symptoms being endorsed.

Gambling problems typically begin in adolescence or early adulthood, (Petry et al., 2005; Blanco et al., 2015) which is a crucial time when individuals are developing close friendships, attempting to complete academic studies, and making decisions about longer term life goals. (Cohen et al., 2003) An improved understanding of gambling in young people, across the full range of diagnostic symptoms, may shed light on causal mechanisms but also facilitate early interventions to avert the progression of disease over time. The relative lack of longitudinal studies in young people designed to explore gambling behavior transitions has been highlighted by other researchers. (Sagoe et al., 2017) Latent class growth analysis (a form of growth mixture modeling) is a statistical approach that accounts both for how individuals change over time but also how they cluster into homogenous groups with respect to growth trajectories This approach has been used with success to investigate various mental health symptoms e.g. (Witkiewitz et al., 2013; Borsboom et al., 2016) but has received only limited application in the context of gambling problems.

Longitudinal research has suggested that gambling may tend to reduce over time in young people, though there is considerable heterogeneity. (Edgerton et al., 2015) Initial longitudinal work using latent class modeling found there to be three classes: consistent non-gambling, consistent non-risk gambling, and risky-and-problem gambling. (Sagoe et al., 2017) The identification of data-driven subtypes of disordered gambling, using a rich set of information provided by structured clinical interview, collected over time, may constitute a valuable first step for public health and neuroscience research. Therefore, the aims of the current study were to (i) identify distinct subtypes of gambling based on latent class modeling of longitudinal data from the Structured Clinical Interview for Gambling Disorder (SCI-GD); and (ii) to profile these subtypes in terms of demographic, clinical, and cognitive measures.

## 2 Method

### 2.1 Participants

Study participants were recruited using media advertisements, which asked “Do you gamble?” Media advertisements were in newspapers, and using physical adverts in public places, in a large US city. The inclusion criteria were being aged 18–29 years, being non-treatment seeking, and having gambled at least five times in the past year. Subjects were excluded if they were unable to give informed consent, or were unable understand/undertake the study procedures. The study was ethically approved by Institutional Review Boards (University of Chicago, and University of Minnesota). All participants provided informed consent, and all procedures were carried out in accordance with the Declaration of Helsinki. Participants were compensated with a $50 gift card for a local department store for taking part in the study. They attended the study site (an academic research center) to undertake a detailed clinical assessment, questionnaires, and cognitive tasks. All clinical assessments were undertaken by individuals fully trained in the use of these instruments, under the direct supervision of a board certified psychiatrist specializing in the assessment and treatment of impulsive and compulsive disorders. Participants were then contacted for annual follow-up visits over the subsequent three years.

### 2.2 Clinical assessments

Demographic information collected by the interviewer included age, gender, ethnicity, level of education, body mass index (BMI), alcohol consumption (times/week), smoking (packs per day equivalent), and whether the individual had a family history of addiction in one or more first-degree relatives. Education level was scored as: 1 = Less than high school, 2 = High school graduate/General Education Degree (GED), 3 = Some College, 4 = College Graduate, 5 = Advanced/Professional Degrees (College+). Family history of addiction was defined as first-degree relative with history of gambling disorder or substance use disorder. Gambling symptoms were evaluated using the Structured Clinical Interview for (SCI-GD). The SCI-GD comprises a previously extensively validated instrument, the Structured Clinical Interview for Pathological Gambling (SCI-PG),(Grant et al., 2004) updated for DSM-5 (since the SCI-PG was developed using DSM-IV). This approach consisted of removing the criterion “committed illegal acts such as forgery, fraud, theft, or embezzlement to finance gambling regarding illegal acts,” which was present in previous manual, DSM-IV; and reduction of the diagnostic threshold from five to four criteria, consistent with DSM-5. The remaining criteria were unchanged. By convention, endorsement of four or more items on the SCI-GD would indicate Gambling Disorder, while endorsing 1–3 criteria would be considered problem gambling. Participants were also asked about the frequency of gambling behavior as well as money lost gambling in the preceding year, using a timeline follow-back method for gambling. (Weinstock et al., 2004) The Mini-International Neuropsychiatric Interview (Sheehan et al., 1998) was completed to identify mainstream mental disorders (e.g. depression, anxiety, substance use); and the Minnesota Impulse Control Disorder (MIDI) (Grant, 2008) was used to identify impulse control disorders (e.g. compulsive buying disorder, compulsive sexual behavior, hair pulling disorder, skin picking disorder). Both have good-excellent test-retest and interrater reliability. (Sheehan et al., 1998; Chamberlain and Grant, 2018)

### 2.3 Questionnaire assessments

The following questionnaires were completed by participants: the Barratt Impulsivity Questionnaire (BIS-11) (Patton et al., 1995; Stanford et al., 2016) to quantify personality-related impulsiveness in the three factor domains (motor, non-planning, and attentional impulsivity); the Padua inventory (Sanavio, 1988) to comprehensively quantify obsessive-compulsive symptoms; and the Quality of Life Inventory (QOLI). (Frisch et al., 1993) The Barratt and Padua questionnaires were included as they assess their respective phenomena dimensionally and impulsivity-compulsivity is likely to play a role in the presentation of disordered gambling. We included the QOLI, which assesses 16 domains of contentedness and life satisfaction, in order to provide a summary score to evaluate functioning.

### 2.4 Cognitive assessments

Cognitive testing was undertaken in a quiet room, with a trained administrator, using the Cambridge Neuropsychological Test Automated Battery (CANTAB). We focused on domains previously strongly implicated in disordered gambling, namely decision-making, set-shifting, and response inhibition. (Clark, 2010; Grant et al., 2011; van Holst et al., 2010) The number of tasks was limited for pragmatic reasons to avoid participant fatigue; and also to minimize the number of multiple comparisons.

Decision-making was examined using the Cambridge Gamble Task (CGT). (Rogers et al., 1999) Participants were told that for each trial, the computer had hidden a ‘token’ inside one of ten boxes shown on the screen. These boxes were each either red or blue, and the participant indicated whether they felt the token would be hidden behind a red or a blue box. After making this judgment, participants gambled a proportion of their points on whether their color choice was correct. The key outcome measures were (i) mean proportion of points gambled; (ii) quality of decision-making (the proportion of trials where the volunteer chose red when red boxes were in the majority and vice versa – i.e. made the logical color choice); (iii) and risk adjustment (tendency to adjust how many points are gambled depending on the degree of risk).

We assessed response inhibition using the Stop-Signal Task, (Aron et al., 2014) a paradigm in which the participant viewed a series of directional arrows appearing one per time on-screen, and made quick motor responses depending on the direction of each arrow (left button for a left-facing arrow, and vice versa). On a subset of trials, an auditory stop-signal occurred (a ‘beep’) to indicate that response suppression was needed for the given trial. The main outcome measure of the Stop-Signal Task is the stop-signal reaction time, which is an estimate of the time taken by the given volunteer's brain to suppress a response that would normally be undertaken.

Set-shifting was measured using the Intra-Dimensional/Extra-Dimensional Set-shift task (IED). (Pantelis et al., 1999) This task, derived from the Wisconsin Card Sorting Task, quantifies several aspects of rule learning and flexible behavior. Volunteers choose from two stimuli presented on the screen on each trial, and attempt to discover an underlying rule governing which stimulus is ‘correct’ (based on simple feedback provided by the computer). One the volunteer has learnt a given rule, the task then changes the rule. The main outcome measure on the task is the total number of errors made, adjusted for stages that were not attempted.

### 2.5 Data analysis

We used latent class growth analysis which is a form of growth mixture model with constrained within class variances of growth trajectories to zero. Such model is suitable for identification of distinct classes in longitudinal data. (Jung and Wickrama, 2008) For each time-point, measurement model of SCI-GD is specified; in our case, single latent variable comprising the 9 SCI-GD symptoms. These latent variables are then used as growth indicators. To ensure measurement invariance, we constrained factor loadings and item thresholds of SCI-GD items in measurement model to be equal across time-points. Intercepts, slopes and curvature of individual growth trajectories as well as their clustering are then estimated. Latent growth analysis identifies a number of latent classes based on cohesive trajectories. The decision on the final number of classes was based on model fit indices including Akaike Information Criterion, Bayesian Information Criterion and classification entropy. Analysis of variance (ANOVA) was then used to explore baseline demographic, clinical and cognitive differences between the identified longitudinal gambling subtypes.

In follow-up analysis, we used repeated measures ANOVA to examine differences in the trajectories of symptoms over time in the latent classes: latent gambling score was the dependent variable, the within-subject factor was time, and the between-subject factor was latent class.

Statistical significance level was set at α = 0.05 throughout. These analyses were undertaken using JMP Pro and SPSS software. Latent class growth analysis was carried in MPlus 8. (Muthén and Muthén, 2016)

## 3 Results

The total sample comprised 575 individuals (mean age [SD] = 22.3 [3.6] years; 376 (65.4%) male), education level 3.2 (0.8) – indicating participants were typically college educated to some degree. Retention over time was as follows: year 1 = 388 subjects, year 2 = 274 subjects, year 3 = 166 subjects.

**Fig. 1:**
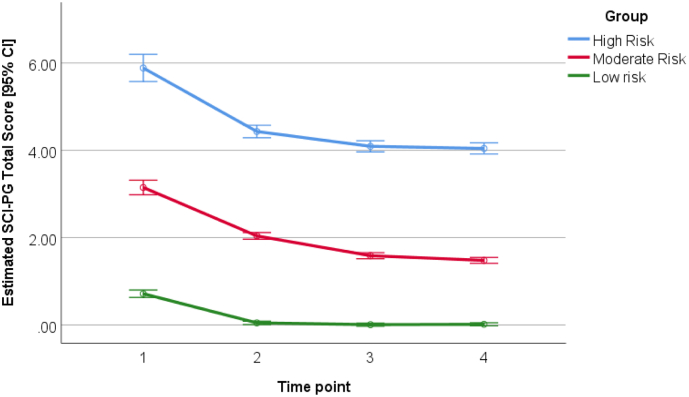
Computed SCI-GD total scores (number of diagnostic items endorsed) over time in each of the three identified latent groups. The Y-axis indicates SCI-GD total items endorsed, derived from latent modeling and fitting baseline data in regression. The X-axis indicates the time-point (baseline, +1 year, + 2 year, and + 3 year).

Latent growth modeling of the SCI-GD across all time-points indicated that the optimal solution had three latent subtypes (Fig. 1). These three latent groups were termed: low harm gamblers (*n* = 431, 75.0% of the sample), intermediate harm gamblers (*n* = 112, 19.5%), and high harm gamblers (*n* = 32, 5.5%).

The baseline demographic and clinical characteristics of the three latent gambling groups are presented in Table 1 and the baseline scores on the questionnaires and cognitive tasks for the three latent gambling groups are shown in Table 2. It can be seen that the groups differed on key demographic, clinical, and cognitive measures. These are considered further in the discussion.

**Table 1:** Baseline demographic and clinical characteristics of longitudinal gambling subtypes. Data are displayed as mean (standard deviation) or number of cases [% of group]. Post hoc tests were conducted when there was a main effect of group for a measure, defined as *p* < .05 Bonferroni corrected for the number of tests (Significant ANOVA *p* values at *p* < .05 Bonferroni corrected for the number of tests in the table are **underlined**) and significant post-hoc tests at *p* < .05 are shown with superscript letters a-c.

	Latent Classification Group Mean (SD) or N [%]			ANOVA, main effect of group	
	Low harm (N = 431)^a^	Intermediate harm (N = 112)^b^	High harm (N = 32)^c^	F (df)	p
Age, years	21.7 (3.4)^b,c^	23.7 (3.5)^a^	24.8 (3.0)^a^	24.02 (2572)	<0.001
SCI-GD total score	0.6 (0.9)^b,c^	2.7 (2.0)^a,c^	7.1 (1.5)^a,b^	534.5 (2572)	<0.001
Sex male, n (%)	279 [64.7%]	77 [68.8%]	20 [62.5%]	0.766#	0.682
Ethnicity caucasian, n [%]	350 [81.4%]^b,c^	57 [50.9%]^a,c^	6 [19.4%]^a,b^	79.822#	<0.001
Education score	3.24 (0.85)	3.16 (0.78)	2.8 (0.95)	3.3965 (2, 572)	0.0341
Quality of life t-score	47.2 (11.1)^b,c^	43.5 (11.0)^a,c^	38.6 (18.9)^a,b^	11.12 (2566)	<0.001
First-degree relative with an addiction, n [%]	110 [25.5%]^b,c^	49 [43.8%]^a^	20 [63.5%]^a^	27.70#	<0.001
Body Mass Index (BMI), kg/m^2^	23.8 (4.5)^b,c^	26.2 (6.5)^a,c^	27.2 (8.0)^a,b^	14.203 (2560)	<0.001
Presence of one or more mainstream mental disorders on MINI, n [%]	125 [29.0%]^b,c^	62 [55.9%]^a^	22 [68.8%]^a^	41.623#	<0.001
Presence of one or more impulse control disorders on MIDI, n [%] [besides gambling disorder]	28 [7.6%]^b,c^	19 [20.0%]^a^	8 [29.6%]^a^	18.444#	<0.001
Amount lost to gambling past year, $	698 (2812)^b,c^	2394 (5186)^a,c^	6258 (6938)^a,b^	39.0 (2571)	<0.001
Number of times alcohol consumed per week	1.3 (1.4)^b,c^	1.5 (1.3)^a^	2.4 (2.1)^a^	8.167 (2569)	<0.001
Nicotine consumption, packs per day equivalent	0.09 (0.24)^b,c^	0.19 (0.33)^a,c^	0.41 (0.53)^a,b^	17.27 (2526)	<0.001

SCI-GD = Structured Clinical Interview for Gambling Disorder; MINI = Mini International Neuropsychiatric Inventory; MIDI = Minnesota Impulse Disorder Inventory. Education Score is rated from 0 (finished education before 16 years of age) to 5 (several higher degrees). For non-parametric variables or where normality was violated, the overall qualitative pattern of significant results was confirmed using equivalent non-parametric tests. # indicates Likelihood Ratio Chi-Square test. Note that degrees of freedom differ for some measures as participants were not mandated to answer all questions, since some questions were of a sensitive nature.

**Table 2:** Baseline personality-related impulsivity, obsessive-compulsive symptoms, and cognitive characteristics of longitudinal gambling subtypes. Data are displayed as mean (standard deviation) or number of cases [% of group]. Post hoc tests were conducted when there was a main effect of group for a measure, defined as *p* < .05 Bonferroni corrected for the number of tests (Significant ANOVA *p* values at *p* < .05 Bonferroni corrected for the number of tests in the table are **underlined**) and significant post-hoc tests at *p* < .05 are shown with superscript letters a-c.

	Latent classification group Mean (SD) or N [%]			ANOVA, main effect of group	
	Low harm (N = 431)^a^	Intermediate harm (N = 112)^b^	High harm (N = 32)^c^	F	p
Personality-related measures					
Barratt impulsivity, attentional	16.7 (4.0)	17.4 (4.2)	17.2 (4.3)	1.317 (2570)	0.269
Barratt impulsivity, motor	23.2 (4.4)^b,c^	25.5 (4.9)^a^	25.5 (5.4)^a^	12.917 (2570)	**<0.001**
Barratt impulsivity, non-planning	23.7 (5.2)^b,c^	25.5 (5.2)^a^	26.0 (6.3)^a^	7.327 (2571)	**<0.001**
Padua compulsivity total score	15.3 (14.0)^b,c^	23.5 (20.7)^a,c^	39.3 (30.6)^a,b^	37.205 (2569)	**<0.001**

Cognitive measures					
CGT Overall proportion of points bet	0.52 (0.14)^b,c^	0.59 (0.13)^a^	0.60 (0.14)^a^	13.936 (2568)	**<0.001**
CGT Quality of decision-making	0.96 (0.08)^b,c^	0.91 (0.10)^a^	0.91 (0.10)^a^	17.2188 (2568)	**<0.001**
CCT Risk adjustment	1.75 (1.19)^b,c^	1.06 (1.08)^a,c^	0.53 (0.99)^a,b^	28.518 (1568)	**<0.001**
SST stop-signal reaction time, msec	176.5 (58.4)^b^	200.0 (73.1)^a^	192.0 (91.4)	6.5085 (2569)	**0.002**
IED Total errors (adjusted)	22.8 (22.8)^b,c^	30.5 (25.9)^a^	35.6 (21.6)^a^	8.371 (2569)	**<0.001**

For non-parametric variables or where normality was violated, the overall qualitative pattern of significant results was confirmed using equivalent non-parametric tests.

Fig. 1 shows computed SCI-GD total scores for the three groups at different time-points. It can be seen that the high harm group had mean 5.9 symptoms endorsed at baseline, and 4.0 at end point (indicating that the typical person had the full disorder that remained at follow-up); the intermediate group endorsed on average 3.1 symptoms at baseline, and 1.5 symptoms at follow-up; and the low harm group endorsed mean 0.7 symptoms at baseline and 0.0 symptoms at follow-up. Repeated measures ANOVA of latent scores indicated a significant main effect of time (F = 499.6, *p* < .001), a significant group x time interaction (F = 3.921, *p* = .001), and a significant main effect of group (F = 2034.0, *p* < .001). This indicates that the latent subtypes differed overall in terms of gambling symptoms, but also differed in the rate of change over time in those symptoms.

Across the whole sample, a total of 27 participants showed worsening of gambling symptoms over three years (4.7% of the sample) based on the scores derived from latent class growth modeling. The numbers and (percentages) of people in each group exhibiting worsening of symptoms over time, according to latent scores, were as follows: low harm 19 (4.4%), intermediate harm 6 (5.4%) and high harm 2 (6.3%).

## 4 Discussion

This study used the data-driven approach of latent class modeling to identify subtypes of gambling symptoms based on trajectories: a high harm group with moderate-severe gambling disorder (in terms of mean symptoms endorsed) who remained symptomatic at follow-up; an intermediate group with problem gambling who reduced their symptom severity over time; and a low harm group who started and stayed asymptomatic during the follow-up period. This study is somewhat in keeping with previous research that has found that problem gambling is perhaps transitory in many individuals,. (Slutske et al., 2003) We found worsening of gambling symptoms over time occurred in around 5% of the sample, and this rate did not differ significantly across the gambling groups. It should be noted that while the three groups differed in their symptom trajectories at a statistical level, the actual mean changes in scores over time were fairly similar between the groups.

The three groups differed significantly in a number of baseline demographic and clinical measures (Table 1). This was due to progression from the low to moderate to high harm groups in terms of: more gambling symptoms endorsed, worse quality of life, higher body mass indices, higher presence of mental disorders, larger amount of money lost to gambling, and higher substance use (alcohol, nicotine). Overall, these results indicate that even intermediate harm levels of gambling symptoms have profound negative associations in terms of mental but also physical health. Furthermore, the gambling groups also differed on personality-related and cognitive measures (Table 2). From low to moderate to high harm groups, there was an increase in levels of impulsivity on the Barratt Impulsivity Scale, and compulsivity on the Padua obsessive-compulsive inventory. Interestingly, the effect on the Barratt Scale was observed for motor and non-planning, but not attentional scores. For the cognitive measures (Table 2), both the moderate and high harm gambling groups showed significant deficits in aspects of decision-making and cognitive flexibility compared to the low harm group. Again, this may indicate that the neurobiological changes linked to disordered gambling are evident relatively early, even in people who have intermediate harm gambling that would not meet current diagnostic thresholds for gambling disorder.

There are several limitations to this study. Some of our participants were under the age of 21 years. As young adults reach the legal gambling age, their gambling behaviors may change because they have legal access to age-restricted venues and the patterns of associations with gambling-related and cognitive variables may also change over time. Another limitation is that, as with any longitudinal study, there is inevitable loss of participants over time due to drop-out. It may be that individuals who show worsening of gambling over time are less likely to be retained in longitudinal studies. The statistical approaches used herein are ideal in such situations because they make maximal use of all available data and allow data for subjects subsequently lost-to-follow-up to still be utilized within the modeling. This can be contrasted for example to use of repeated measures ANOVA without such imputation, which leads to exclusion of data for all subjects who did not complete the full length of the cohort time period. Another limitation is that the findings may not generalize to other settings, such as treatment-seeking individuals, and/or those recruited in clinical rather than community settings. Finally, we assessed subjects annually for convenience. Of course, gambling symptoms may fluctuate in individuals on a shorter time frame; it was not the aim of this study to assess shorter-term fluctuations in gambling symptoms. We present group-level changes over time, measured annually, but this may overlook nuanced or subtle changes that may be observable using more frequent measurements.

Strengths of this study include the large sample size of non-treatment seeking young adults, and the use of latent class growth analysis. This in turn has the potential to drive better primary interventions, as our data indicate that even the intermediate harm gambling group presented with untoward cognitive, mental, and physical health associations. Future work should extend the current latent class analytic approach into clinical settings, as the current study focused on non-treatment seeking individuals. This may ultimately contribute to more meaningful or clinically helpful ways of subclassifying patients for the purposes of treatment selection or prioritization, including for early interventions. Because risk of worsening of symptoms over time was around 5% in this sample, irrespective of group, our findings highlight the need for larger scale longitudinal studies of disordered gambling in order to identify predictive markers.

## Ethical statement

The study was ethically approved by Institutional Review Boards (University of Chicago, and University of Minnesota). All participants provided informed consent, and all procedures were carried out in accordance with the Declaration of Helsinki.

## Funding

This work was supported by a Center of Excellence in Gambling Research grant from the National Center for Responsible Gaming (NCRG) to Dr. Grant (USA); and by a Wellcome Trust Clinical Fellowship to Dr. Chamberlain (UK; Reference 110,049/Z/15/Z). NCRG is an industry-funded not-for-profit institution. Dr. Stochl received support from the NIHR Collaboration for Leadership in Applied Health Research and Care (CLAHRC) East of England (EoE) at the Cambridgeshire and Peterborough NHS Foundation Trust. The views expressed are those of the authors and not necessarily those of the NHS, the NIHR or the Department of Health and Social Care.

## Contributors

Samuel Chamberlain: Dr. Chamberlain undertook statistical analyses and drafted the manuscript.

Jan Stochl: Dr. Stochl undertook statistical analyses and drafted the manuscript.

Jon Grant: Dr. Grant designed the study, collected the data and drafted the manuscript.

All authors had full access to the study data.

## Declaration of Competing Interest

Dr. Chamberlain consults for Cambridge Cognition, Shire, Promentis, and Ieso Digital Health. Dr. Chamberlain receives a stipend for role as Associate Editor at Neuroscience and Biobehavioral Reviews; and for his role as Associate Editor at Comprehensive Psychiatry. Dr. Stochl consults for Ieso Digital Health. Dr. Grant has received research grants from NIMH, National Center for Responsible Gaming, and Forest and Roche Pharmaceuticals. He receives yearly compensation from Springer Publishing for acting as Editor-in-Chief of the Journal of Gambling Studies and has received royalties from Oxford University Press, American Psychiatric Publishing, Inc., Norton Press, and McGraw Hill.
